# Compliance and Kinetostatics of a Novel 2PRS-2PSS Compliant Parallel Micromanipulator: Modeling and Analysis

**DOI:** 10.3390/mi15040526

**Published:** 2024-04-14

**Authors:** Jun Ren, Hui Jiang

**Affiliations:** Hubei Key Laboratory of Modern Manufacturing Quantity Engineering, School of Mechanical Engineering, Hubei University of Technology, Wuhan 430068, China; 102100014@hbut.edu.cn

**Keywords:** compliant parallel micromanipulator, flexure hinge, compliance modeling, kinetostatic modeling

## Abstract

A novel 2PRS-2PSS (P represents the prismatic pair, R represents the revolute hinge, S represents the spherical hinge) compliant parallel micromanipulator with two translational DOFs and two rotational DOFs is presented, and its compliance model and kinetostatic model are sequentially developed and analyzed. Initially, an analytical model used to describe the compliance of this micromanipulator was developed using the compliance matrix method (CMM). Through a comparison with finite element analysis, the accuracy of this analytical model is confirmed, and the influence of various dimensional and structural parameters on the compliance behavior is investigated. Subsequently, the micromanipulator is treated as an equivalent spring system, allowing for the derivation of its governing equation based on the established compliance model. From this equation, a kinetostatic model relating input forces to output displacements is derived. Validation of this model is performed by comparing analytical results with finite element simulations under specific motion trajectories, revealing a maximum relative error of 6.18%. This close agreement verifies the accuracy of the kinetostatic model. Finally, the impact of the parameters of the flexure hinge on the mapping matrix is examined to offer insights into minimizing undesired displacements, providing valuable guidance for optimizing the micromanipulator’s performance.

## 1. Introduction

In recent years, fields such as biology, medicine, and industrial assembly have been moving toward smaller operating objects and higher precision requirements. Examples include cell injection, microsurgery, and fiber optic docking. There is an increasing demand for micromanipulators with excellent performance [1,2]. Traditional rigid manipulators, due to issues like clearance, friction, and backlash in their mechanisms, have made it difficult to meet the high-precision requirements of micromanipulation systems. In contrast, micromanipulators based on compliant parallel mechanisms utilize the deformation of flexure hinges to transmit motion, effectively addressing these issues. They also offer advantages such as high stiffness and compact structure, enabling high-precision manipulations [3,4,5,6,7,8]. Therefore, it has attracted a large number of scholars to conduct research on micromanipulators based on compliant parallel mechanisms with various configurations.

Compliance plays a crucial role in the design and analysis of compliant parallel mechanisms, as it serves as a key performance metric. The study of the kinematics and dynamics of compliance mechanisms relies on the foundation provided by the compliance model [9,10,11]. Zhang et al. [12] conducted research on an integrated compliant redundant parallel mechanism for the XY micro-adjustment of photolithography projection lens optical components. They employed the compliance matrix method to calculate the compliance of individual limbs within the mechanism, including output compliance, input compliance, and the input–output ratio of the system. Li et al. [13] proposed a novel piezoelectric-driven XY micro-motion platform. They used the compliance matrix method to establish the compliant model of the mechanism and conducted static and dynamic analyses. Mishra et al. [9] examined a 6-DOF serial-parallel compliant micromanipulator built on a Stewart platform featuring a displacement amplification structure. By applying the compliance matrix method, they developed a compliance model for the mechanism and investigated the impact of various parameters on its compliance. Ren et al. [14] proposed the design of a novel *n*-4R-compliant parallel micro-pointing mechanism. They utilized the compliance matrix method to develop a compliance model for the mechanism and investigated how different parameters affected the compliance model.

In addition to compliance, kinetostatics is another indispensable area of research for compliant parallel mechanisms. The kinetostatic model of these mechanisms serves as the foundation for motion control and significantly aids in studying the reduction of parasitic displacement within the mechanism. It is widely acknowledged that compliant parallel mechanisms offer advantages such as high precision, the absence of friction, the absence of backlash, and a compact structure due to the utilization of flexure hinges. However, analyzing the kinetostatics in a compliant parallel mechanism presents greater challenges compared to a rigid one, primarily due to the inherent relationship between kinematics and the elasticity in flexure hinges. This coupling necessitates a comprehensive modeling approach that extends beyond traditional kinematics or statics analysis [15]. In the past few decades, researchers have devised numerous techniques and methodologies to model the kinetostatics model of compliant mechanisms, such as the pseudo-rigid-body model, Castigliano’s second theorem, compliance matrix method, elastic beam theory, etc. [16]. Midha et al. [17] analyzed the fixed-oriented compliant beam with an inflection point by utilizing the concept of a pseudo-rigid-body model and improved the calculation accuracy by setting the inflection point. Ni et al. [18] established the kinematic, static, and dynamic models of the compliant piezoelectric micro-cracker based on the three-stage amplification by using the pseudo-rigid-body model method. Wadikhaye et al. [19] analyzed the input and output stiffness of the serial motion XYZ scanner by utilizing Castigliano’s second theorem and estimated the first natural frequency and the travel range of the scanner using the input–output stiffness. Dong et al. [20] developed a bridge amplifier and established a motion-statics model based on the compliance matrix method and a dynamics model based on the Lagrange method for the mechanism. Wang et al. [21] developed a kinetostatic model utilizing the Cosserat rod theory. The proposed model aimed to minimize positioning errors resulting from external loads. Ren et al. conducted a series of studies on compliant parallel mechanisms. In one study [14], they designed an *n*-4R-compliant micro-pointing mechanism and developed its kinetostatic model using the compliance matrix method. They investigated the impact of parameters on the kinetostatic mapping matrix. In a subsequent study [22], Ren et al. designed a novel 3PSS/S-compliant micro-turntable. They established the kinetostatic model of the mechanism using both the compliance matrix method and the pseudo-rigid-body model method. The two models were then compared in terms of accuracy. More recently, Ren et al. [23] introduced a generalized 3-PSS-compliant parallel micro-motion platform. They focused on investigating the compliance and kinetostatic models of this platform. Ling et al. [24] proposed a semi-analytical matrix displacement method for modeling the kinetostatics of compliant mechanisms. This method is particularly useful for complex compliant mechanisms that comprise serial-parallel substructures. Based on the work of Ling et al., Arredondo-Soto et al. [25,26] combined various methods from other literature to derive a more general approach for kinetostatic modeling. They used this approach to establish the relationship between input force/displacement and the displacement of the mobile platform for a 3-RRR spherical-compliant parallel mechanism.

Currently, there is limited research on 4-DOF-compliant parallel mechanisms. The 4-DOF parallel mechanism offers broader application possibilities compared to mechanisms with fewer degrees of freedom (less than 4-DOF). Simultaneously, the 4-DOF parallel mechanism features a simpler structure and lower control complexity compared to its 6-DOF counterpart. Therefore, this paper presents a novel 4-DOF 2PRS-2PSS compliant parallel micromanipulator. The compliance model and kinetostatic model of the micromanipulators are sequentially developed and analyzed. Firstly, the compliance matrix of a single flexure hinge is computed, followed by deriving the compliance of the PSS branch and PRS branch separately. Subsequently, the overall compliance model of the mechanism is constructed using the compliance matrix method, and its accuracy is verified through finite element simulation. The study then investigates the effects of variations in both the dimensional parameters of the mechanism and the structural parameters of the flexure hinges on the overall compliance. Secondly, the mechanism is simplified as an equivalent spring system. The governing equation of this equivalent spring system is derived from the previously established compliant model. By utilizing this governing equation, the kinetostatic model of the mechanism is formulated. The accuracy of the kinetostatic model is validated by comparing analytical calculations with finite element simulations of the specified motion trajectory of the mechanism. Finally, the impact of the structural parameters of the flexure hinges on the mapping matrix of the kinetostatic model is analyzed.

## 2. Structure of 2PRS-2PSS Compliant Parallel Micromanipulator

**Figure 1:**
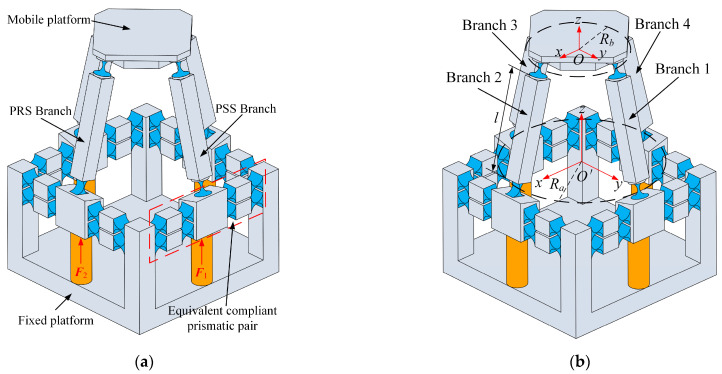
(**a**) Structure of the micromanipulator; (**b**) structural parameters and coordinate systems of the micromanipulator.

The 2PRS-2PSS compliant parallel micromanipulator is a 4-DOF micromanipulation platform with two translational and two rotational degrees of freedom. As shown in Figure 1a, it consists of a mobile platform, a fixed platform, two symmetrically arranged PRS branches, and two symmetrically arranged PSS branches. The links of each branch are connected to the mobile platform through flexure spherical hinges. The links of each PRS branch are connected to the prismatic pair through flexure revolute hinges, while the links of each PSS branch are connected to the prismatic pair through flexure spherical hinges. To enhance the compliance and adaptability of the mechanism, an equivalent compliant prismatic pair consisting of eight flexure revolute hinges is employed. The flexure revolute hinges that connect to the link have the same structure as the eight flexure revolute hinges, but their structural parameters are different.

The structural parameters and coordinate system of the mechanism are set as shown in Figure 1a. The length of the links is denoted as *l*. The radius of the circle formed by the rotation centers of the four hinges connected to the equivalent compliant prismatic pair is defined as the fixed platform radius, *R_a_*, with the center of the circle denoted as *O*’. The radius of the circle formed by the rotation centers of the four hinges connected to the mobile platform is defined as the mobile platform radius, *R_b_*, with the center of the circle denoted as *O*. The assumption is made that the radius of a fixed platform is always larger than that of the mobile platform. The mechanism utilizes four piezoelectric ceramics for actuation. By controlling the inputs of these actuators, the mobile platform can generate movement along the *x* and *z* axes and rotation around the *x* and *y* axes. Therefore, the micro-manipulator can achieve position adjustments in the *x* and *z* directions during operation, while also having the capability to adjust the orientation angles around the *x* and *y* axes simultaneously.

## 3. Modeling and Analysis of the Compliance of the 2PRS-2PSS Compliant Parallel Micromanipulator

The organization of this section is as follows. Firstly, the compliance model of the micromanipulator is developed using the compliance matrix method. Secondly, the compliance model is validated through finite element simulation to ensure its accuracy. Finally, the impact of some structural parameters on the overall compliance of the micromanipulator is analyzed.

### 3.1. Compliance Matrix Method

The compliance matrix method considers the flexure hinge as a compliant element while treating other parts as rigid bodies. During the process of compliance modeling, the flexure hinge is regarded as a multi-dimensional hinge, and the overall compliance model of the mechanism is obtained through coordinate transformations. Therefore, the compliance matrix method offers higher accuracy and wider applicability.

Under the condition that one end of the flexure hinge is fixed, deformation will be generated by exerting the forces and moments at the free end of the flexure hinge. Under the assumption of small elastic deformations, the principle of linear superposition holds, meaning that the deformation of the flexure hinge in each direction can be calculated by summing the individual deformations caused by each applied force or moment. Assuming the forces and moments are represented as ***F*** = [*f_x_*, *f_y_*, *f_z_*, *m_x_*, *m_y_*, *m_z_*]^T^, and the corresponding linear displacements and angular displacements are represented as ***X*** = [*δ_x_*, *δ_y_*, *δ_z_*, *θ_x_*, *θ_y_*, *θ_z_*]^T^, the compliance matrix of a single flexure hinge is defined as ***C***. The relationship between the input forces and displacements at the end of the hinge is defined as follows [27]:

(1) X=CF

### 3.2. Compliance Matrix of Single Flexure Hinge or Compliant Element

Before calculating the overall compliance of the micromanipulator, the compliance of a single flexure hinge used in the mechanism should be calculated first. The 2PRS-2PSS compliant parallel micromanipulator employs flexure spherical hinges and flexure revolute hinges, so the compliance of these two types of hinges needs to be calculated separately. Additionally, the equivalent prismatic pair is treated as a compliant element, and its compliance is calculated through the compliance matrix method.

**Figure 2:**
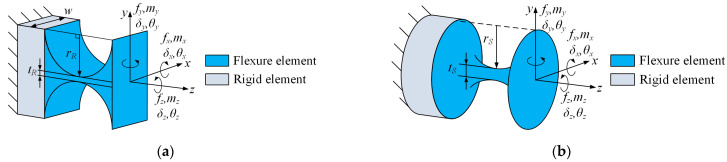
(**a**) Structure parameters of the right-circular flexure revolute hinge; (**b**) structure parameters of the right-circular flexure spherical hinge.

Figure 2a illustrates the structural parameters and coordinate frame configuration of the flexure revolute hinge. In this figure, *r_R_*, *w,* and *t_R_* represent the cutting radius, width, and minimum thickness of the flexure hinge, respectively. Based on its structural characteristics, it can be observed that this hinge exhibits significant compliance in the rotation around the *x*-axis and can be used as a single-degree-of-freedom revolute hinge. The compliance matrix of the flexure revolute hinge can be expressed as Equation (2) [25], and the formulas for calculating the compliance matrix ***C****_R_* can be found in Appendix A.

The structure parameters and coordinate frame setting of the flexure spherical hinge are shown in Figure 2b, where *r_S_* and *t_S_* represent the cutting radius and minimum thickness of the flexure hinge, respectively. It has three degrees of freedom for rotation around the *x*, *y*, and *z* axes and can be used as a universal joint. The compliance matrix of this hinge is denoted as ***C****_S_* and its representation method is consistent with Equation (2), and the formulas for calculating the compliance matrix ***C****_S_* can be found in Appendix A.

(2) $$C_{R}=\left[\begin{array}{cccccc} C_{\delta_{x},f_{x}} & 0 & 0 & 0 & C_{\delta_{x},m_{y}} & 0\\ 0 & C_{\delta_{y},f_{y}} & 0 & C_{\delta y,m_{x}} & 0 & 0\\ 0 & 0 & C_{\delta_{z},f_{z}} & 0 & 0 & 0\\ 0 & C_{\theta_{x},f_{y}} & 0 & C_{\theta_{x},m_{x}} & 0 & 0\\ C_{\theta_{y},f_{x}} & 0 & 0 & 0 & C_{\theta_{y},m_{y}} & 0\\ 0 & 0 & 0 & 0 & 0 & C_{\theta_{z},m_{z}} \end{array}\right]$$

**Figure 3:**
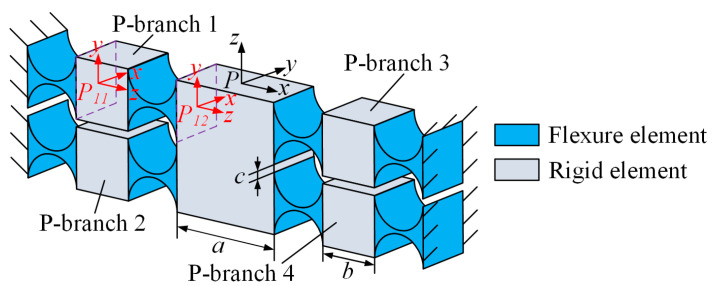
Structure parameters of the equivalent compliant prismatic pair.

The structure of the equivalent compliant prismatic pair is shown in Figure 3. The prismatic pair consists of four identical branches, each of which includes two flexure revolute hinges. These branches are named P-branch *i*. The flexure revolute hinges used here have the same structure as described in Figure 2a but with different dimensional parameters. The cutting radius, width, and minimum thickness of the flexure revolute hinge used here are expressed as *r_P_*, *w_P,_* and *t_P_*, respectively. The structural parameters *a*, *b* and *c* in the equivalent compliant prismatic pair represent the distance of the symmetrical branch, the distance of the adjacent branch and the length of the connecting rod connecting the two flexure revolute hinges on a single branch, respectively. To facilitate the subsequent modeling of overall compliance, the compliance of the equivalent compliant prismatic pair is modeled at coordinate system *P*-*xyz* to form local compliant elements. Thus, the compliance of P-branch 1 and 2 can be calculated as follows:

(3) $$C_{Pbm}^{P}=\sum_{n=1}^{2}T_{Pmn}^{P}C_{RPmn}^{P}{\left(T_{Pmn}^{P}\right)}^{T}\;,\;m=1,2$$

(4) $$T_{Pmn}^{P}=\left[\begin{array}{cc} R_{Pmn}^{P} & R_{Pmn}^{P}P_{Pmn}^{P}\\ 0 & R_{Pmn}^{P} \end{array}\right]$$

where $T_{Pmn}^{P}$ is an adjoint transformation matrix that represents the transformation of the compliance matrix from the local coordinate system, *P_mn_*-*xyz*, to the reference coordinate system, *P*-*xyz*. *m* represents the branch number, and *n* represents the position of the hinge on the branch. Defining the rotation transformation matrix, $R_{Pmn}^{P}$, and the position transformation matrix, $P_{Pmn}^{P}$, their specific expressions are as follows:

(5) $$R_{Pmn}^{P}=\left[\begin{array}{ccc} c\gamma c\beta & c\gamma s\beta s\alpha -s\gamma c\alpha & c\gamma s\beta c\alpha +s\gamma s\alpha \\ s\gamma c\beta & s\gamma s\beta s\alpha +c\gamma c\alpha & s\gamma s\beta c\alpha -c\gamma s\alpha \\ -s\gamma & c\gamma s\alpha & c\gamma c\alpha \end{array}\right],\;P_{Pmn}^{P}=\left[\begin{array}{ccc} 0 & h_{3} & -h_{2}\\ -h_{3} & 0 & h_{1}\\ h_{2} & -h_{1} & 0 \end{array}\right]$$

where *α*, *β*, and *γ* represent the angle of rotation around the *x*, *y,* and *z* axes, respectively. s and c represent sin and cos, respectively. The elements *h*_1_, *h*_2,_ and *h*_3_ in $P_{Pmn}^{P}$ represent the positions of local coordinates in the reference coordinate system; their coordinate form is ***h*** = (*h*_1_, *h*_2_, *h*_3_). The parameters of the adjoint matrix of the coordinate transformation in the prismatic pair are constructed as shown in Table 1.

According to the principles of stiffness and compliance superposition in series-parallel hybrid mechanisms [27], it is known that the overall compliance of the equivalent prismatic pair at the coordinate *P*-*xyz* is as follows:

(6) $$C_{P}={\left({\left(\sum_{m=1}^{4}C_{PBm}^{P}\right)}^{-1}\right)}^{-1}$$

The structure and structural parameters of the local branch are similar, so the compliance of P-branch 3 and P-branch 4 in the reference coordinate system *P*-*xyz* can be obtained by rotating the compliance of P-branch 1 and P-branch 2. The compliance matrix is as follows:

(7) $$\left\{\begin{array}{l} C_{PB3}^{P}=T_{1}^{3}C_{PB1}^{P}{\left(T_{1}^{3}\right)}^{T}\\ C_{PB4}^{P}=T_{2}^{4}C_{PB2}^{P}{\left(T_{2}^{4}\right)}^{T} \end{array}\right.,\;T_{1}^{3}=T_{2}^{4}=\left[\begin{array}{cc} R_{z,\pi } & \\ & R_{z,\pi } \end{array}\right]$$

### 3.3. Compliance Modeling of Single Branch

**Figure 4:**
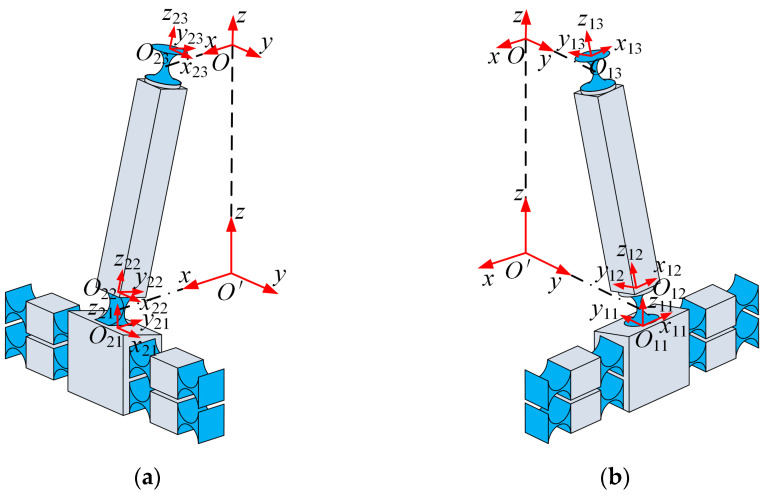
The structure of the branch and the setting of the local coordinate system (**a**) branch 1; (**b**) branch 2.

In general, it is meaningful to discuss the compliance of a compliant mechanism only when it is uniformly discussed in the same coordinate system [27]. Since the 2PRS-2PSS compliant parallel micromanipulator consists of PRS and PSS branches, the compliance of a single branch should be calculated first. The structures of branch 1 and branch 2 are shown in Figure 4. The compliance of the flexure hinge needs to be unified into the reference coordinate system first. Taking branch 1 (PSS branch) and branch 2 (PRS branch) as an example, the conversion Equation (8) describes the process of unifying the compliance of the flexure hinge on the branch to the reference coordinate system *O*-*xyz*.

(8) $$\left\{\begin{array}{l} C_{P_{11}}^{O}=T_{O_{11}}^{O}C_{P_{11}}^{O_{11}}{\left(T_{O_{11}}^{O}\right)}^{T}\\ C_{S_{12}}^{O}=T_{O_{12}}^{O}C_{S_{12}}^{O_{12}}{\left(T_{O_{12}}^{O}\right)}^{T}\\ C_{S_{13}}^{O}=T_{O_{13}}^{O}C_{P_{13}}^{O_{13}}{\left(T_{O_{13}}^{O}\right)}^{T} \end{array},\;\right.\left\{\begin{array}{l} C_{P_{21}}^{O}=T_{O_{21}}^{O}C_{P_{21}}^{O_{21}}{\left(T_{O_{21}}^{O}\right)}^{T}\\ C_{R_{22}}^{O}=T_{O_{22}}^{O}C_{R_{22}}^{O_{22}}{\left(T_{O_{22}}^{O}\right)}^{T}\\ C_{S_{23}}^{O}=T_{O_{23}}^{O}C_{S_{23}}^{O_{23}}{\left(T_{O_{23}}^{O}\right)}^{T} \end{array}\;\right.$$

where $T_{Oij}^{O}$ is the transformation matrix of 6 × 6, which shows that the compliance matrix is transformed from the local coordinate system, *O_ij_*-*x_ij_y_ij_z_ij_*, to the global coordinate system, *O*-*xyz*. The rotation transformation matrix is denoted as $R_{Oij}^{O}$, and the translation transformation matrix is denoted as $P_{Oij}^{O}$. Subscript *i* represents the number of branches in the mechanism, and *j* represents the position index of the flexure hinge or compliance element on the branch, following the numbering rules shown in Figure 4.

When the flexure hinges or compliant elements are connected in series, the compliance is superimposed [27]. Therefore, the compliance of branch 1 and branch 2 in the reference coordinate system can be obtained as follows:

(9) $$\left\{\begin{array}{l} C_{B1}^{O}=C_{P_{11}}^{O}+C_{S_{12}}^{O}+C_{S_{13}}^{O}\\ C_{B2}^{O}=C_{P_{21}}^{O}+C_{S_{22}}^{O}+C_{S_{23}}^{O} \end{array}\right.$$

### 3.4. Compliance Model of the Micromanipulator

According to the principles of stiffness and compliance superposition in series-parallel hybrid mechanisms [27], the compliance of the 2PRS-2PSS compliant parallel micromanipulator can be determined as follows:

(10) $$C_{2\mathrm{PRS}-2\mathrm{PSS}}={\left({\left(\sum_{i=1}^{4}C_{Bi}^{O}\right)}^{-1}\right)}^{-1}$$

where ***C***_2PRS-2PSS_ represents the total compliance of the 2PRS-2PSS compliant parallel micromanipulator, and $C_{Bi}^{O}$ denotes the compliance of each individual branch within the reference coordinate system.

Due to the symmetry of the mechanism, the compliance matrix of branch 3 and branch 4 can be easily obtained by performing coordinate transformations on the compliance matrix of branch 1 and branch 2, as shown in Equation (11).

(11) $$\left\{\begin{array}{l} C_{PB3}^{P}=T_{1}^{3}C_{PB1}^{P}{\left(T_{1}^{3}\right)}^{T}\\ C_{PB4}^{P}=T_{2}^{4}C_{PB2}^{P}{\left(T_{2}^{4}\right)}^{T} \end{array}\right.,\;T_{1}^{3}=T_{2}^{4}=\left[\begin{array}{cc} R_{z,\pi } & \\ & R_{z,\pi } \end{array}\right]$$

where $T_{1}^{3}$ and $T_{2}^{4}$ are the adjoint transformation matrix and ***R****_z,π_* is the rotation transformation matrix, indicating that the compliance matrix rotates 180° around the *z*-axis of the reference coordinate system, *O*-*xyz*. The related parameters of the adjoint matrix of the overall compliance coordinate transformation of the mechanism are listed in Table 2. In this table, *θ* represents the angle between the links and the *z*-axis of the reference coordinate system, and its value can be expressed as follows:

(12) $$\theta =\arcsin\left(\frac{R_{a}-R_{b}}{l+2r_{S}}\right)$$

### 3.5. Verification of Effectiveness of the Compliance Model

To validate the accuracy of the compliance model, finite element analysis is conducted in this section. Table 3 outlines the dimensional parameters of the mechanism, as well as the structural parameters of the flexure hinges utilized. The total compliance can be expressed by Equation (13). Substituting the parameters (listed in Table 3) into Equation (10), one can obtain the analytical results of the compliance of the 2PRS-2PSS compliant parallel micromanipulator.

(13) $$C_{2\mathrm{PRS}-2\mathrm{PSS}}=\left[\begin{array}{cccccc} C_{\delta_{x},f_{x}} & 0 & 0 & 0 & C_{\delta_{x},m_{y}} & 0\\ 0 & C_{\delta_{y},f_{y}} & 0 & C_{\delta y,m_{x}} & 0 & 0\\ 0 & 0 & C_{\delta_{z},f_{z}} & 0 & 0 & 0\\ 0 & C_{\theta_{x},f_{y}} & 0 & C_{\theta_{x},m_{x}} & 0 & 0\\ C_{\theta_{y},f_{x}} & 0 & 0 & 0 & C_{\theta_{y},m_{y}} & 0\\ 0 & 0 & 0 & 0 & 0 & C_{\theta_{z},m_{z}} \end{array}\right]$$

The compliance model of the 2PRS-2PSS compliant parallel micromanipulator underwent validation using the commercial software ANSYS 19.2, affirming the precision of the analytical findings. To optimize computational efficiency and accuracy, the flexure hinge was discretized into tetrahedral meshes with a size of 0.5 mm, while the remaining components were segmented into tetrahedral meshes with a size of 3 mm. The material parameters pertaining to the flexure hinge are listed in Table 4.

By comparing the compliance elements on the main diagonal between the analytical results and the finite element results, the relative errors are calculated and summarized in Table 5. The results show that the relative errors for the six main compliances of the compliance matrix are all below 7.2%. This high level of consistency indicates the accuracy of the established theoretical compliance model. The discrepancies between the analytical and finite element results can be attributed to the following factors: (1) errors in the compliance model of individual flexure hinges; and (2) theoretical modeling is carried out based on the assumption that all components except for the flexure hinges are rigid. However, in the finite element simulation, these ‘rigid’ components simulated their rigid behavior by being set with larger stiffness values. Thus, even if their stiffness is set to be sufficiently large, they may still undergo slight deformations.

### 3.6. Analysis of Compliance Performance of 2PRS-2PSS compliant Parallel Micromanipulator

When designing compliant parallel mechanisms, it is necessary to consider various performance indicators such as compliance, stiffness, and accuracy, etc. And different application scenarios have different requirements for these performances. For applications that require strong adaptability and sensitivity to external disturbances, greater compliance is beneficial. However, for applications that demand high precision and stiffness, it is necessary to control compliance to ensure the stability and accuracy of the mechanism. Therefore, analyzing the influence of parameters in the mechanism on compliance is essential [28].

**Figure 5:**
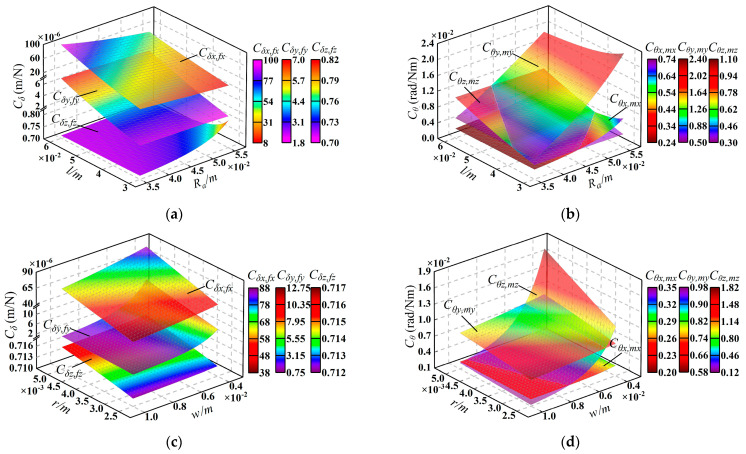

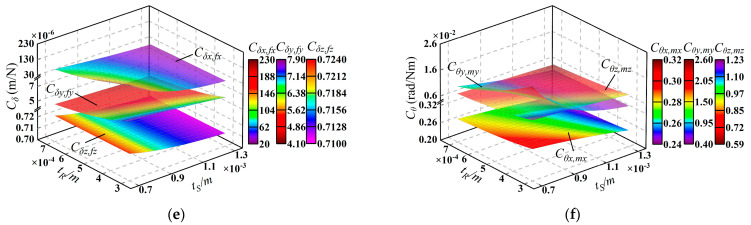
Variation of compliance with the parameters: (**a**) *C_δ_* with *l* and *R_a_*; (**b**) *C_θ_* with *l* and *R_a_*; (**c**) *C_δ_* with *r* and *w*; (**d**) *C_θ_* with *r* and *w*; (**e**) *C_δ_* with *t_R_* and *t_S_*; (**f**) *C_θ_* with *t_R_* and *t_S_*.

In this section, the compliance model established in Section 3.4 is used to analyze the compliance of the micromanipulator. Here, *C_δ_* and *C_θ_* are defined as translational compliance and rotational compliance, respectively. The parameters related to compliance and their variation ranges are listed in Table 6, and parameters *r_S_* and *r_R_* are always the same, so we unified their symbols as *r*. The variation in overall compliance with these parameters is shown in Figure 5.

From Figure 5, we can draw the following conclusions: (1) in translational compliance *C_δ_*, *C_δx_*_,*fx*_ is always the largest and most affected by parameter changes, while *C_δz_*_,*fz*_ is always the smallest. At the same time, in rotational compliance *C_θ_*, *C_θx_*_,*mx*_ is always smaller than *C_θy_*_,*my*_. (2) The structural parameters of the flexure hinges have a more significant influence on *C_δ_* and *C_θ_* compared to the dimensional parameters of the mechanism. Consequently, when designing the mechanism, it is suggested that the structural parameters of the flexure hinges be adjusted with priority to achieve the desired compliance in the micromanipulator.

**Figure 6:**
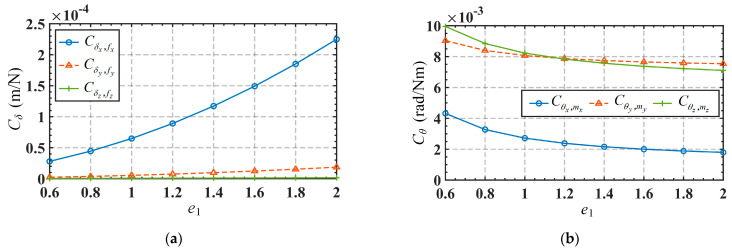
Compliance variations in terms of the scale coefficient *e*_1_: (**a**) *C_δ_*; (**b**) *C_θ_*.

**Figure 7:**
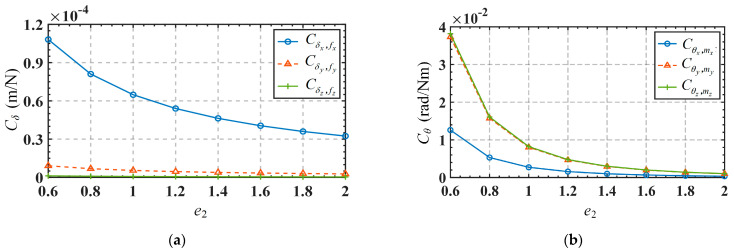
Compliance variations in terms of the scale coefficient *e*_2_: (**a**) *C_δ_*; (**b**) *C_θ_*.

Apart from the influence of these mentioned parameters, it is sometimes necessary to understand the way the micromanipulator compliance scales with the defining geometric diameters. Given the utilization of flexure hinges in the mechanism, there are two possible scaling scenarios: scaling only the dimensional parameters of the mechanism (represented by the scaling coefficient *e*_1_) or simultaneously scaling both the dimensional parameters of the mechanism and the flexure hinge parameters (represented by the scaling coefficient *e*_2_). The variations of the mechanism’s compliance with the scaling coefficients *e*_1_ and *e*_2_ are shown in Figure 6 and Figure 7, respectively. It can be observed from Figure 6 that *C_δ_* and *C_θ_* are directly and inversely correlated with the scaling coefficient *e*_1_, respectively. Comparatively, *C_δx_*,*_fx_* is greatly affected, *C_δy_*_,*fy*_ is less affected, while *C_δz_*_,*fz*_ is almost unaffected, as shown in Figure 6a. It is also noted from Figure 6b that the overall decrease of *C_θx_*_,*mx*_, *C_θy_*_,*my*_, and *C_θz_*_,*mz*_ is generally gradual. It can be seen from Figure 7 that both *C_δ_* and *C_θ_* are inversely correlated with the scaling coefficient *e*_2_. And Figure 7a exhibits the same feature as Figure 6a in that *C_δx_*_,*fx*_ is greatly affected, *C_δy_*_,*fy*_ is less affected, while *C_δz_*_,*fz*_ is almost unaffected. Compared to Figure 6b, *C_θ_* in Figure 7b decreases more sharply in the range with smaller proportion coefficients.

## 4. Kinetostatic Modeling of 2PRS-2PSS compliant Parallel Micromanipulator

The force–displacement relationship at the center point of the end-effector platform can be easily obtained by the compliance model. However, the input force and output displacement of 2PRS-2PSS compliant parallel micromanipulation exist in different coordinate systems. Therefore, it is of vital necessity to establish the kinetostatic model of this micromanipulation.

### 4.1. Kinetostatic Modeling under Single Input Force

**Figure 8:**
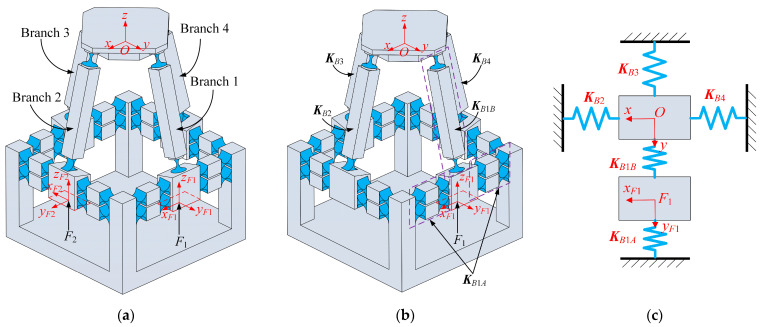
(**a**) Branch number and force loading position; (**b**) simplification of equivalent stiffness with force ***F***_1_ acting alone; (**c**) equivalent spring system.

To achieve the output displacements of the mobile platform, four input forces are exerted at the center point of the bottom surface of the equivalent prismatic pairs, as shown in Figure 8a. The local coordinate systems are established at this center point, allowing us to obtain the local generalized input forces as follows: ***F****_i_* = [*f_i_*_,*x*_, *f_i_*_,*y*_, *f_i_*_,*z*_, *m_i_*_,*x*_, *m_i_*_,*y*_, *m_i_*_,*z*_], *i* = 1, 2, 3, 4. The value of *i* denotes system *F_i_*-*x_Fi_y_Fi_z_Fi_*. The displacement of the center point of the mobile platform relative to the global coordinate system *O*-*xyz* is expressed as follows: ***U***_2PRS-2PSS_ = [*δ_x_*, *δ_y_*, *δ_z_*, *θ_x_*, *θ_y_*, *θ_z_*]. Under the assumption of linear deformations, the force–displacement mapping relationship of the mechanism can be determined using the principle of superposition.

To facilitate the kinetostatic modeling, we simplify the 2PRS-2PSS compliant parallel micromanipulator as an equivalent spring system, as presented in Figure 8b,c. Note that the concept of equivalent stiffness can also be found in [27,29]. Since the same branches of the mechanism are symmetrically arranged, only branch 1 and branch 2 are taken for analysis. The input forces applied to these two branches are denoted as ***F***_1_ and ***F***_2_, respectively. Correspondingly, the resulting displacements are ***U***_1_ and ***U***_2_, respectively. Then, the elastic deformation of the system can be described by the governing equation (Equation (14)) based on Hooke’s law. The stiffness matrices in Equation (14) are computed by Equation (15). It should be noted that the stiffness matrix in Equation (15) can be calculated by using Equations (8) and (10), as shown in Equations (18) and (19).

(14) $$\left[\begin{array}{cc} {\left(K_{OO}\right)}_{F_{i}} & K_{OF_{i}}\\ K_{F_{i}O} & K_{F_{i}F_{i}} \end{array}\right]\left[\begin{array}{c} U_{i}\\ U_{F_{i}} \end{array}\right]=\left[\begin{array}{c} F_{O}\\ F_{i} \end{array}\right]$$

(15) $$\left\{\begin{array}{l} {\left(K_{OO}\right)}_{F_{i}}=K_{BiB}^{O}+{\left(C_{2\mathrm{PRS}-2\mathrm{PSS}}\right)}^{-1}-K_{Bi}\\ K_{F_{i}F_{i}}=K_{BiA}^{F_{i}}+K_{BiB}^{F_{i}}\\ K_{OF_{i}}=-{\left(T_{O}^{O}\right)}^{-T}K_{BiB}^{O}{\left(T_{O}^{F_{i}}\right)}^{-1}\\ K_{F_{i}O}=-{\left(T_{O}^{F_{i}}\right)}^{-T}K_{BiB}^{O}{\left(T_{O}^{O}\right)}^{-1} \end{array}\right.$$

where

(16) $$T_{O}^{O}=I_{6\times 6},\;T_{O}^{F_{i}}=\left[\begin{array}{cc} R_{O}^{F_{i}} & P_{O}^{F_{i}}T_{O}^{F_{i}}\\ 0_{3\times 3} & R_{O}^{F_{i}} \end{array}\right]$$

where

(17) $$P_{O}^{F_{i}}=\left[\begin{array}{ccc} 0 & -z_{i} & -y_{i}\\ z_{i} & 0 & x_{i}\\ y_{i} & -x_{i} & 0 \end{array}\right],\;R_{O}^{F_{i}}=I_{3\times 3},\;i=1,2$$

(18) $$\left\{\begin{array}{l} K_{B1A}^{O}={\left(C_{P_{11}}^{O}\right)}^{-1},\;K_{B1B}^{O}={\left(C_{S_{12}}^{O}+C_{S_{13}}^{O}\right)}^{-1}\\ K_{B2A}^{O}={\left(C_{P_{21}}^{O}\right)}^{-1},\;K_{B2B}^{O}={\left(C_{R_{22}}^{O}+C_{S_{23}}^{O}\right)}^{-1} \end{array}\right.$$

(19) $$\left\{\begin{array}{l} K_{BiA}^{F_{i}}={\left(T_{O}^{F_{i}}{\left(K_{BiA}^{O}\right)}^{-1}{\left(T_{O}^{F_{i}}\right)}^{T}\right)}^{-1}\\ K_{BiB}^{F_{i}}={\left(T_{O}^{F_{i}}{\left(K_{BiB}^{O}\right)}^{-1}{\left(T_{O}^{F_{i}}\right)}^{T}\right)}^{-1} \end{array}\right.,\;i=1,2$$

In Equation (15), the superscripts *O* and *F_i_* associated with each stiffness matrix denote that the stiffness matrix is defined with respect to the coordinate frames *O*-*xyz* and *F_i_*-*x_Fi_y_Fi_z_Fi_*, respectively. In Equations (16) and (17), the symbol ***I*** represents the unit matrix, and *x_i_*, *y_i_*, and *z_i_* denote the position coordinates of the local coordinate system *F_i_*-*x_Fi_y_Fi_z_Fi_* in the reference coordinate system.

Since there is no force applied to the mobile platform, ***F****_O_* in Equation (14) can be set to 0, which yields the following:

(20) $$U_{i}=C_{TOF_{i}}\cdot F_{i},\;i=1,2$$

where

(21) $$C_{TOF_{i}}=-{\left({\left(K_{OO}\right)}_{F_{i}}-K_{OF_{i}}K_{F_{i}F_{i}}^{-1}K_{F_{i}O}\right)}^{-1}\left(K_{OF_{i}}K_{F_{i}F_{i}}^{-1}\right),\;i=1,2$$

Then, the kinetostatic model of this micromanipulator under a single input force is obtained by Equation (20).

### 4.2. Kinetostatic Modeling of 2PRS-2PSS compliant Parallel Micromanipulator

Define the displacement of the center of the mobile platform as ***U***_2PRS-2PSS_. Based on the principle of superposition, displacement of ***U***_2PRS-2PSS_ resulting from the combined exertions of forces ***F***_1_, ***F***_2_, ***F***_3,_ and ***F***_4_ can be considered as the superposition of the displacements ***U***_1_, ***U***_2_, ***U***_3,_ and ***U***_4_ generated by ***F***_1_, ***F***_2_, ***F***_3,_ and ***F***_4_ acting alone. Therefore, the displacement of ***U***_2PRS-2PSS_ can be expressed as follows:

(22) $$U_{2\mathrm{PRS}-\mathrm{PSS}}=\sum_{i=1}^{4}U_{i}$$

According to Equations (20) and (22), one can then obtain the kinetostatic model of a 2PRS-2PSS compliant parallel micromanipulator, as follows:

(23) $$U_{2\mathrm{PRS}-\mathrm{PSS}}=\sum_{i=1}^{4}C_{TOF_{i}}\cdot F_{i}$$

where the matrix, $C_{TOF_{i}}$, represents the mapping relationship between the force, ***F****_i_*, and the displacement, ***U****_i_* (*i* = 1, 2, 3, 4).

Since branches 3 and 4 are symmetrically arranged with branches 1 and 2, respectively, $C_{TOF_{3}}$ and $C_{TOF_{4}}$ can be easily obtained through the rotational transformation of $C_{TOF_{1}}$ and $C_{TOF_{2}}$, respectively.

(24) $$C_{TOF_{i+2}}={\left[T_{\pi }\right]}^{-T}\left[C_{TOF_{i}}\right],\;T_{\pi }=\left[\begin{array}{cc} R_{z,\pi } & 0_{3\times 3}\\ 0_{3\times 3} & T_{z,\pi } \end{array}\right]$$

where the matrix $C_{TOF_{i+2}}$ (*i* = 1, 2) describes the relationship between the input forces ***F****_i_*_+2_ (*i* = 1, 2) and the corresponding output displacements ***U****_i_*_+2_ (*i* = 1, 2). And ***R****_z_*_,*π*_ represents the coordinate transformation matrix with 180° rotation around the *z*-axis of the coordinate system *O*-*xyz*.

## 5. Verification and Analysis of the Kinetostatic Model

In Section 4, the kinetostatic model of the 2PRS-2PSS compliant parallel micromanipulator has been established. Here, we validate the accuracy of the established model through a comparative analysis between theoretical calculations and finite element simulations, utilizing an example. Additionally, we investigate the impact of variations in the structural parameters of the flexure hinges on the kinetostatic model. It is important to note that the parameters in this example are consistent with those presented earlier in Section 3.5.

### 5.1. Numerical Calculation and Simulation Analysis of the Kinetostatic Model

The 2PRS-2PSS compliant parallel micro-manipulator is a 4-DOF mechanism. Therefore, the elements related to the movements of four functional directions of the mechanism in the model are extracted from Equation (23), and the simplified kinetostatic model is obtained as in Equation (25).

(25) $$\left[\begin{array}{c} \delta_{x}\\ \delta_{z}\\ \theta_{x}\\ \theta_{y} \end{array}\right]=\left[\begin{array}{cccc} C_{\delta_{x},f_{1,z}} & C_{\delta_{x},f_{2,z}} & C_{\delta_{x},f_{3,z}} & C_{\delta_{x},f_{4,z}}\\ C_{\delta_{z},f_{1,z}} & C_{\delta_{z},f_{2,z}} & C_{\delta_{z},f_{3,z}} & C_{\delta_{z},f_{4,z}}\\ C_{\theta_{x},f_{1,z}} & C_{\theta_{x},f_{2,z}} & C_{\theta_{x},f_{3,z}} & C_{\theta_{x},f_{4,z}}\\ C_{\theta_{y},f_{1,z}} & C_{\theta_{y},f_{2,z}} & C_{\theta_{y},f_{3,z}} & C_{\theta_{y},f_{4,z}} \end{array}\right]\left[\begin{array}{c} f_{1,z}\\ f_{2,z}\\ f_{3,z}\\ f_{4,z} \end{array}\right]$$

The mapping matrix in Equation (25) is composed of the elements of the 1st, 3rd, 4th, and 5th rows, and the 3rd column of the matrix $C_{TOF_{i}}$, and it is defined as $C_{TOFrc}$. And *f*_1,*z*_, *f*_2,*z*_, *f*_3,*z*_, and *f*_4,*z*_, respectively, denote the components of forces ***F***_1_, ***F***_2_, ***F***_3_, and ***F***_4_ along the *z*-axis of the local coordinate system. Substituting the parameters in Table 3 into $C_{TOFrc}$ yields the following:

(26) $$C_{TOFrc}^{An}=\left[\begin{array}{cccc} 0 & -1.61\times 10^{-6} & 0 & 1.61\times 10^{-6}\\ 8.87\times 10^{-6} & 8.93\times 10^{-6} & 8.87\times 10^{-6} & 8.93\times 10^{-6}\\ 6.73\times 10^{-5} & 0 & -6.73\times 10^{-5} & 0\\ 0 & -5.36\times 10^{-5} & 0 & 5.36\times 10^{-5} \end{array}\right]$$

**Figure 9:**
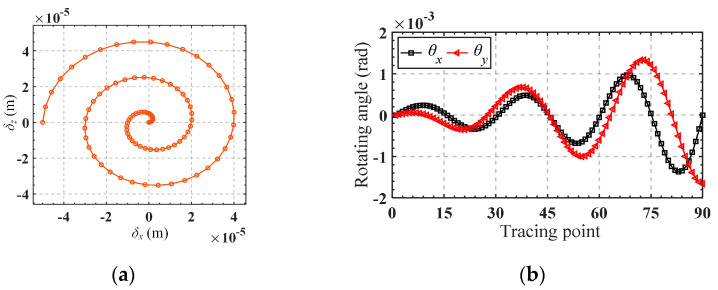
(**a**) The given moving trajectory; (**b**) the given rotational trajectory.

**Figure 10:**
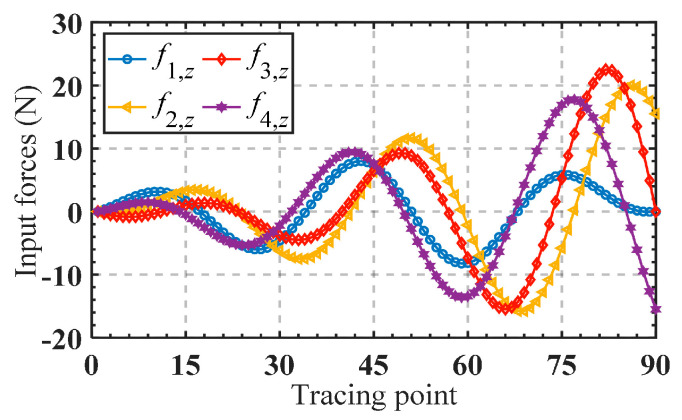
Curves of input forces *f*_1,*z*_, *f*_2,*z*_, *f*_3,*z*_ and *f*_4,*z*_.

It can be observed from the matrix that the elements of the first row and the fourth row are proportional, which means that the output displacement *δ_x_* and *θ_y_* are always proportional in value (but their units are different). To facilitate the calculation of input forces from given output displacements, we extract the first three rows of the matrix, $C_{TOFrc}^{An}$, to form a new matrix, $C_{TOFrc(r1,2,3)}^{An}$. Obviously, Matrix $C_{TOFrc(r1,2,3)}^{An}$ has full column rank, so the input forces can be determined by taking the right inverse of $C_{TOFrc(r1,2,3)}^{An}$ as shown in Equation (27). The trajectory equation is given by Equations (28) and (29), and the motion trajectory is shown in Figure 9. The obtained input forces are shown in Figure 10.

(27) $$\left[\begin{array}{c} f_{1,z}\\ f_{2,z}\\ f_{3,z}\\ f_{4,z} \end{array}\right]={\left(C_{TOFrc\left(r1,2,3\right)}^{An}\right)}^{T}{\left(C_{TOFrc\left(r1,2,3\right)}^{An}\cdot {\left(C_{TOFrc\left(r1,2,3\right)}^{An}\right)}^{T}\right)}^{-1}\left[\begin{array}{c} \delta_{x}\\ \delta_{z}\\ \theta_{x} \end{array}\right]$$

(28) $$\left\{\begin{array}{l} \delta_{x}=L\cos\left(10000L\pi \right)\\ \delta_{z}=L\sin\left(10000L\pi \right) \end{array}\right.,\;0\leq L\leq 5\times 10^{-5}$$

(29) $$\left\{\begin{array}{l} \theta_{x}=2e^{\left(t/3\right)}\sin\left(3t\right)\times 10^{-4},\;0\leq t\leq 2\pi \\ \theta_{y}=\left(C_{\theta_{y},f_{2,z}}/C_{\delta_{x},f_{2,z}}\right)\delta_{x} \end{array}\right.$$

**Figure 11:**
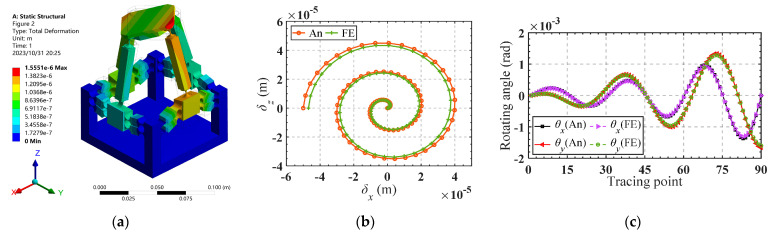
(**a**) FE model; (**b**) analytical and FE moving trajectories; (**c**) analytical and FE rotational trajectory.

**Figure 12:**
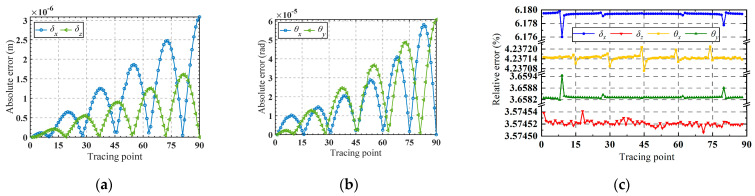
(**a**) The absolute error of *δ_x_* and *δ_z_* in the moving trajectory; (**b**) the absolute error of *θ_x_* and *θ_y_* in the rotational trajectory; (**c**) the relative error of the motion trajectory.

Substituting the obtained input forces into the finite element software for simulation, the corresponding trajectory can be obtained, as shown in Figure 11a. Figure 11b,c presents a comparison between the analytical trajectories and the finite element trajectories. Good consistency of the trajectories demonstrates the accuracy of the established kinetostatic model. The absolute error between the theoretical analysis and the finite element analysis for the motion trajectory is illustrated in Figure 12a,b. It is shown that the absolute errors of the moving trajectory and rotation trajectory are directly correlated with the radius of the spiral trajectory and the rotating angle, respectively.

It can be observed from Figure 12c that the maximum relative error occurs in the *x*-axis movement direction, stabilizing between 6.176% and 6.180%. The minimum error occurs in the *z*-axis movement direction, stabilizing between 3.57450% and 3.57454%. The relative error for rotation around the *x*-axis ranges from 3.6582% to 3.6594%, while for rotation around the *y*-axis, it ranges from 4.23708% to 4.23730%. Good consistency indicates the effectiveness of the established kinetostatic model.

### 5.2. Analysis of the Influence of Parameters on the Kinetostatic Model

In Section 4, the kinetostatic model of the 2PRS-2PSS compliant parallel micromanipulator was established, and its correctness was verified in Section 5.1. Since the micromanipulator generates parasitic displacement during its motion, it affects the positioning accuracy of the micromanipulator. Therefore, in this section, an analysis of the influence of the structural parameters of the flexure hinges on the mapping matrix is further conducted, which provides a reference on how to reduce parasitic displacement.

**Figure 13:**
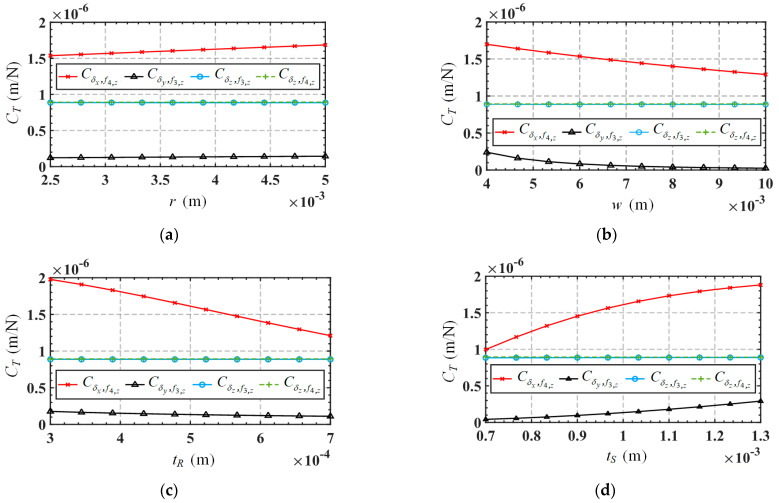
Variation of *C_T_* in terms of the structure parameters: (**a**) *r*; (**b**) *w*; (**c**) *t_R_*; (**d**) *t_S_*.

**Figure 14:**
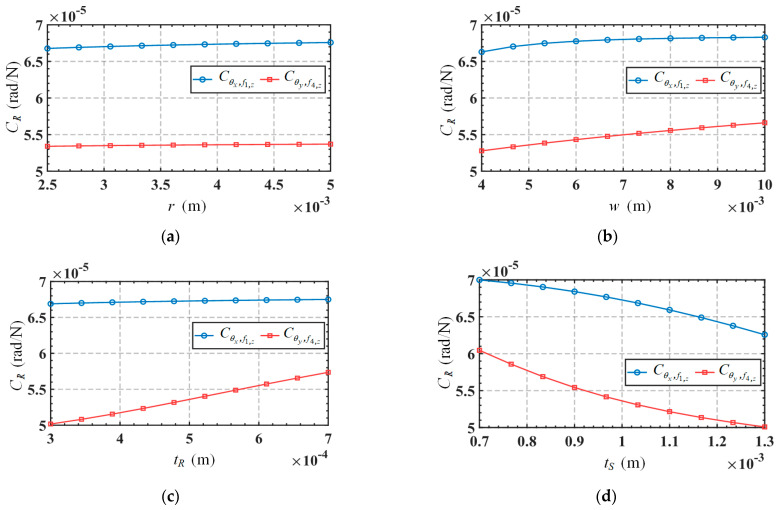
Variation of *C_R_* in terms of the structure parameters: (**a**) *r*; (**b**) *w*; (**c**) *t_R_*; (**d**) *t_S_*.

Taking into account the movements in non-functional directions (*δ_y_* and *θ_z_*), the relationship between the input forces and output displacements of the mechanism can be expressed as Equation (30), and the mapping matrix in the equation is defined as ***C***_A_. Substitute the parameters in Table 3 into the mapping matrix ***C****_A_* to obtain the results as shown in Equation (31). The element in matrix ***C****_A_* is defined as the compliance coefficient. It can be seen from the mapping matrix that the mechanism will produce parasitic displacement moving along the *y*-axis direction. For convenience, the first three and last three rows of the mapping matrix are defined as the translation-related mapping matrix *C_T_* and rotation-related mapping matrix *C_R_*, respectively. Meanwhile, since the structural symmetry of the mechanism, some of the compliance coefficients in the mapping matrix are the same in absolute values (with the positive or negative sign only indicating the direction of motion). Therefore, we only need to analyze the compliance coefficients $C_{\delta_{x},f_{4,z}}$, $C_{\delta_{y},f_{3,z}}$, $C_{\delta_{z},f_{3,z}}$, $C_{\delta_{z},f_{4,z}}$, $C_{\theta_{x},f_{1,z}}$ and $C_{\theta_{y},f_{3,z}}$, where $C_{\delta_{y},f_{3,z}}$ is the compliance coefficient related to parasitic displacement. Figure 13 shows the variations of the compliance coefficient *C_T_* in terms of the parameters *r*, *w*, *t_R,_* and *t_S_*. Variations of the compliance coefficient *C_R_*, in terms of these parameters, are shown in Figure 14. The range of variation for these parameters is listed in Table 6.

(30) $$\left[\begin{array}{c} \delta_{x}\\ \delta_{y}\\ \delta_{z}\\ \theta_{x}\\ \theta_{y}\\ \theta_{z} \end{array}\right]=\left[\begin{array}{cccc} C_{\delta_{x},f_{1,z}} & C_{\delta_{x},f_{2,z}} & C_{\delta_{x},f_{3,z}} & C_{\delta_{x},f_{4,z}}\\ C_{\delta_{y},f_{1,z}} & C_{\delta_{y},f_{2,z}} & C_{\delta_{y},f_{3,z}} & C_{\delta_{y},f_{4,z}}\\ C_{\delta_{z},f_{1,z}} & C_{\delta_{z},f_{2,z}} & C_{\delta_{z},f_{3,z}} & C_{\delta_{z},f_{4,z}}\\ C_{\theta_{x},f_{1,z}} & C_{\theta_{x},f_{2,z}} & C_{\theta_{x},f_{3,z}} & C_{\theta_{x},f_{4,z}}\\ C_{\theta_{y},f_{1,z}} & C_{\theta_{y},f_{2,z}} & C_{\theta_{y},f_{3,z}} & C_{\theta_{y},f_{4,z}}\\ C_{\theta_{z},f_{1,z}} & C_{\theta_{z},f_{2,z}} & C_{\theta_{z},f_{3,z}} & C_{\theta_{z},f_{4,z}} \end{array}\right]\left[\begin{array}{c} f_{1,z}\\ f_{2,z}\\ f_{3,z}\\ f_{4,z} \end{array}\right]$$

(31) $$C_{A}^{\mathrm{An}}=\left[\begin{array}{cccc} 0 & -1.61\times 10^{-6} & 0 & 1.61\times 10^{-6}\\ -1.34\times 10^{-7} & 0 & 1.34\times 10^{-7} & 0\\ 8.87\times 10^{-7} & 8.93\times 10^{-7} & 8.87\times 10^{-7} & 8.93\times 10^{-7}\\ 6.73\times 10^{-5} & 0 & -6.73\times 10^{-5} & 0\\ 0 & -5.36\times 10^{-5} & 0 & 5.36\times 10^{-5}\\ 0 & 0 & 0 & 0 \end{array}\right]$$

From Figure 13, we can draw the following conclusions: (1) The parasitic displacement-related compliance coefficient $C_{\delta_{y},f_{3,z}}$ is always much smaller than the mobile functional-direction-related compliance coefficients ($C_{\delta_{x},f_{4,z}}$, $C_{\delta_{z},f_{3,z}}$ and $C_{\delta_{z},f_{4,z}}$), indicating that the mechanism has a higher motion accuracy. (2) The parasitic displacement-related compliance coefficient $C_{\delta_{y},f_{3,z}}$ is directly correlated to the parameter *t_S_*, and inversely correlated to the parameter *w*, while it is not sensitive to the variations in parameters *r* and *t_R_*. Therefore, reducing parameter *t_S_* and increasing parameter *w* are preferable measures to reduce parasitic displacement. However, such changes to these parameters will also decrease the movement in the functional direction, so designers need to make reasonable trade-offs based on actual requirements. It is worth noting that changing the parameters to reduce parasitic displacement will also affect the rotation functional-direction-related compliance coefficients ($C_{\theta_{x},f_{1,z}}$ and $C_{\theta_{y},f_{3,z}}$), as shown in Figure 14. From Figure 14, we can draw the following conclusions: (1) The rotation functional-direction-related compliance coefficients, $C_{\theta_{x},f_{1,z}}$, are inversely correlated to the parameter *t_S_*, while not significantly affected by the changes of parameters *r*, *w*, and *t_R_*. (2) Another rotation functional-direction-related compliance coefficient $C_{\theta_{y},f_{3,z}}$ is directly correlated to the parameters *w* and *t_R_*, and inversely correlated to the parameter *t_S_*, while not sensitive to the variation of parameter *r*.

## 6. Conclusions

A novel 2PRS-2PSS compliant parallel micromanipulator is presented, and its compliance model and kinetostatic model are successively established and analyzed. The correctness of the two models is confirmed through FE simulation. The conclusions are as follows:

(1) In the verification of the compliance model, comparison results show that the maximum relative errors of the elements on the main diagonal of the compliance model between the theoretical calculation and the finite element analysis do not exceed 7.2%, indicating the correctness of the compliance model. Analysis of the effect of the parameters on the compliance model indicates that the structural parameters of the flexure hinges have a more significant influence on the compliance of the micromanipulator compared to the dimensional parameters of the mechanism.
(2) By equating the 2PRS-2PSS micromanipulator to a spring system, the kinetostatic model of the micromanipulator is established based on the previously established compliance model according to Hooke’s law. In the validation of the kinetostatic model, comparison results show that the maximum relative errors between the theoretical analysis and the finite element analysis are within 6.18%, confirming the accuracy of the kinetostatic model.
(3) In the analysis of the effect of the parameters on the kinetostatic model, it is found that compared to other parameters, the width (*w*) of the flexure revolute hinge and the cutting thickness (*t_S_*) of the flexure spherical hinge has the most significant influence on the parasitic displacement. Adjusting these parameters by increasing *w* and reducing *t_S_* can effectively minimize the parasitic displacement of the mechanism, thereby improving motion accuracy.

It is worth noting that when adjusting parameters to reduce the parasitic displacement of the micromanipulator, the overall compliance of the micromanipulator also changes. On the one hand, increasing the width (*w*) of flexure revolute hinges is advantageous for reducing parasitic displacement, but it also decreases overall compliance. On the other hand, reducing the minimum cutting thickness (*t_S_*) of flexure spherical hinges can also reduce parasitic displacement but increase overall compliance. All parameter adjustment schemes aimed at reducing parasitic displacement will to some extent decrease the working range of the micromanipulator because the impact of parameters on the compliance coefficient is universal and consistent. For micromanipulators, reducing parasitic displacement is beneficial for improving accuracy, and when external disturbances (i.e., external forces acting on the mobile platform) are significant, reducing overall compliance also helps improve accuracy. When external disturbances during micromanipulator operation are significant, lower overall compliance (i.e., higher overall stiffness) is beneficial. As long as the mechanism’s working range is within the allowed range, increasing the width of flexure revolute hinges can reduce parasitic displacement and increase the overall compliance of the mechanism, thereby improving operational precision. However, when external disturbances during the micromanipulator operation are minimal or negligible, it is possible to simultaneously increase the width of flexure revolute hinges and decrease the minimum cutting thickness of flexure spherical hinges to reduce parasitic displacement while meeting the working range requirements of the micromanipulator.

## Figures and Tables

**Table 1:** Parameters of the transformation matrix in the equivalent prismatic pair.

Transformation Matrix	*h*_1_	*h*_2_	*h*_3_	*α*	*β*	*γ*
$T_{P11}^{P}$	−0.5a	0	$-\left(r_{P}+0.5t_{P}\right)$	π/2	0	π/2
$T_{P12}^{P}$	$-\left(0.5a+b+2r_{P}\right)$	0	$-\left(r_{P}+0.5t_{P}\right)$	π/2	0	π/2
$T_{P21}^{P}$	−0.5a	0	$-\left(3r_{P}+1.5t_{P}+c\right)$	π/2	0	π/2
$T_{P22}^{P}$	$-\left(0.5a+b+2r_{P}\right)$	0	$-\left(3r_{P}+1.5t_{P}+c\right)$	π/2	0	π/2

**Table 2:** Parameters of the transformation matrix in the mechanism.

Transformation Matrix	*x*	*y*	*z*	*α*	*β*	*γ*
$T_{P11}^{P}$	0	$R_{a}+r_{S}\sin\theta$	$-\left(l+3r_{S}\right)\cos\theta$	0	0	*π*
$T_{P12}^{P}$	0	$R_{a}+r_{S}\sin\theta$	$-\left(l+3r_{S}\right)\cos\theta$	−*θ*	0	*π*
$T_{P21}^{P}$	0	$R_{a}+r_{S}\sin\theta$	$r_{S}\cos\theta$	−*θ*	0	*π*
$T_{P22}^{P}$	$R_{a}+r_{S}\sin\theta$	0	$-\left(l+3r_{S}\right)\cos\theta$	0	0	*π*/2
$T_{P22}^{P}$	$R_{a}-r_{S}\sin\theta$	0	$-\left(l+3r_{S}\right)\cos\theta$	−*θ*	0	*π*/2
$T_{P22}^{P}$	$R_{b}-r_{S}\sin\theta$	0	$r_{S}\cos\theta$	−*θ*	0	*π*/2

**Table 3:** Structural parameters of 2PRS-2PSS compliant parallel micromanipulator.

Item	Values (mm)	Item	Values (mm)	Item	Values (mm)
*R_a_*	40	*r_R_*	3.75	*c*	1
*R_b_*	25	*t_R_*	0.5	*r_P_*	3.75
*l*	52.5	*w*	5	*t_P_*	0.5
*r_S_*	3.75	*a*	20	*w_P_*	8
*t_S_*	1	*b*	10		

**Table 4:** Material parameters of the flexure hinge.

Type of Hinge	Material	Density (kg/m^3^)	Young’s Modulus (GPa)	Poisson Ratio
Spherical hinge	CuBe_2_	8000	128	0.3
Revolute hinge	65 Mn	8000	206	0.3

**Table 5:** Error comparison of overall compliance.

Compliance	An	FE	Relative Error
$C_{\delta_{x},f_{x}}$ (m/N)	0.00006483	0.00006668	2.77%
$C_{\delta_{y},f_{y}}$ (m/N)	0.00000545	0.00000587	7.13%
$C_{\delta_{z},f_{z}}$ (m/N)	0.00000072	0.00000069	3.43%
$C_{\theta_{x},m_{x}}$ (rad/N)	0.00272024	0.00263453	3.25%
$C_{\theta_{y},m_{y}}$ (rad/N)	0.00806618	0.00805665	0.12%
$C_{\theta_{z},m_{z}}$ (rad/N)	0.00822998	0.00864432	4.79%

**Table 6:** Variation range in the parameters of the micromanipulator.

Item	Variation Range (mm)	Item	Variation Range (mm)
*l*	30~60	*w*	4~10
*R_a_*	35~55	*t_R_*	0.3~0.7
*r* (*r_S_, r_R_*)	2.5~5	*t_S_*	0.7~1.3

## Data Availability

Data are contained within the article.

## Appendix A

The formulas for calculating the compliance matrix of the right-circular flexure revolute hinge are as follows (*w*, *r_R_*, *t_R_*):

(A1) $$\left\{\begin{array}{l} C_{\delta_{x},f_{x}}=\frac{12}{Ew^{3}}\int_{0}^{2r_{R}}\frac{x^{2}}{t^{2}(x)}dx+\frac{1}{Gw}\int_{0}^{2r_{R}}\frac{1}{t(x)}dx\\ C_{\delta_{y},f_{y}}=\frac{12}{Ew}\int_{0}^{2r_{R}}\frac{x^{2}}{t^{3}(x)}dx\\ C_{\delta_{z},f_{z}}=\frac{1}{Ew}\int_{0}^{2r_{R}}\frac{1}{t(x)}dx\\ C_{\theta_{x},m_{x}}=\frac{12}{Ew}\int_{0}^{2r_{R}}\frac{1}{t^{3}(x)}dx \end{array},\;\left\{\begin{array}{l} C_{\theta_{y},m_{y}}=\frac{12}{Ew^{3}}\int_{0}^{2r_{R}}\frac{1}{t(x)}dx\\ C_{\delta_{x},m_{y}}=C_{\theta_{y},f_{x}}=\frac{12}{Ew^{3}}\int_{0}^{2r_{R}}\frac{x}{t(x)}dx\\ C_{\delta_{y},m_{x}}=C_{\theta_{x},f_{y}}=\frac{12}{Ew}\int_{0}^{2r_{R}}\frac{x}{t^{3}(x)}dx \end{array}\right.\right.$$

where $t(x)=t_{R}+2r_{R}-2\sqrt{x(2r_{R}-x)},\;(x\in \left[0,2r_{R}\right])$, *E*, and *G* are the Young’s modulus and shear modulus of the material, respectively.

The formulas for calculating the compliance matrix of the right-circular flexure spherical hinge are as follows [30] (*r_S_*, *t_S_*):

(A2) $$\left\{\begin{array}{l} C_{\delta_{x},f_{x}}=\frac{64\int_{0}^{2r_{S}}\frac{x^{2}}{t^{4}(x)}dx+\kappa (1+\upsilon )\int_{0}^{2r_{S}}\frac{1}{t^{2}(x)}dx}{\pi E}\\ C_{\delta_{y},f_{y}}=C_{\delta_{x},f_{x}}\\ C_{\delta_{z},f_{z}}=\frac{4}{\pi E}\int_{0}^{2r_{S}}\frac{1}{t^{2}(x)}dx\\ C_{\theta_{x},m_{x}}=\frac{64}{\pi E}\int_{0}^{2r_{S}}\frac{1}{t^{4}(x)}dx\\ C_{\theta_{y},m_{y}}=C_{\theta_{x},m_{x}}\\ C_{\theta_{z},m_{z}}=(1+\upsilon )C_{\theta_{x},m_{x}}\\ C_{\delta_{x},m_{y}}=C_{\theta_{y},f_{x}}=r_{S}C_{\theta_{x},m_{x}}\\ C_{\delta_{y},m_{x}}=C_{\theta_{x},f_{y}}=-C_{\delta_{x},m_{y}} \end{array}\right.$$

where $t(x)=t_{S}+2r_{S}-2\sqrt{x(2r_{S}-x)},\;(x\in \left[0,2r_{S}\right])$, *κ* is the correction coefficient; here, we take 10/9, *υ* is the Poisson’s ratio. *E* is Young’s modulus of the material.
